# Per- and Polyfluoroalkyl Substances (PFAS) in Michigan: A Novel High-Throughput Method with Liquid Chromatography Tandem Mass Spectrometry (LC-MS/MS) Analysis of 39 PFAS Compounds in Human and Bovine Dried Blood Spots (DBS)

**DOI:** 10.3390/toxics14090753

**Published:** 2026-08-26

**Authors:** Jessica M. Morrison, Sarah Y. Lockwood-O’Brien, Chelsea A. Bielicki, Douglas J. Carmack, Julia R. Feeley, Timothy A. Karrer, Matthew J. Geiger

**Affiliations:** Bureau of Laboratories, State of Michigan Department of Health and Human Services, 3350 North Martin Luther King Junior Boulevard, Lansing, MI 48906, USA; lockwoodobriens@michigan.gov (S.Y.L.-O.); bielickic@michigan.gov (C.A.B.); carmackd@michigan.gov (D.J.C.); feeleyj2@michigan.gov (J.R.F.); karrert@michigan.gov (T.A.K.); geigerm@michigan.gov (M.J.G.)

**Keywords:** fluorocarbons, biological monitoring, dried blood spots, environmental monitoring, liquid chromatography–mass spectrometry, analytical sample preparation methods

## Abstract

This study presents a novel method for analyzing per- and polyfluoroalkyl substances (PFAS) in dried blood spots (DBS), employing a hybrid solvent-based and matrix-matched calibration curve, minimal sample volume, and high-throughput preparation. This process produces a concentrated, purified sample, which is analyzed using liquid chromatography coupled with tandem mass spectrometry (LC-MS/MS). As part of method validation, key performance metrics such as accuracy, precision, sensitivity, carryover, specificity, interferences, and maximum dilution capability were assessed. The method quantifies 39 PFAS compounds across various classes, achieving limits of detection in the low ng L^−1^ range, with most analytes having a limit of quantitation below the first calibrator (50 ng L^−1^). The total method accuracy exceeded 81%, surpassing the performance goal of ≥70%, while method imprecision was below 28%, outperforming the goal of ≤30%. Specificity, as measured by a normalized percent matrix bias, was below 11%, with limited and minimal matrix effects observed. Interference from bile acids was determined to be insignificant at biologically relevant concentrations. Over the past three years, this method has been successfully utilized in biomonitoring studies of Michigan residents, demonstrating its robustness and efficiency.

## 1. Introduction

The ubiquity of per- and polyfluoroalkyl substances (PFAS) inextricably intertwines environmental and public health challenges. With its abundant freshwater resources, Michigan faces significant challenges regarding PFAS contamination in its water bodies, posing threats to both aquatic ecosystems and human health alike. Additionally, the legacy of industrial activities as well as the presence of military bases and firefighting training activities have contributed to the widespread distribution of PFAS across the state. The presence of PFAS in everyday life by way of consumer products further exacerbates the issue and exposure pathways. Amidst numerous routes of exposure to PFAS, blood products are a vital medium for gauging chronic PFAS exposure levels and interpreting their impact on public health. Dried blood spots (DBS) are an often overlooked yet key piece of the PFAS exposure puzzle. In this study, we introduce a novel analytical method designed to address this issue by way of DBS in human and bovine blood, utilizing liquid chromatography tandem mass spectrometry (LC-MS/MS). This manuscript details the confirmed accuracy/recovery, precision, reportable range verification, sensitivity, method carryover, analytical specificity, interferences/cross-reactivity, and maximum dilution for a high-throughput method for quantitating the levels of a wide spectrum of 39 PFAS compounds.

Exposure to PFAS can occur through a multitude of pathways, including ingestion of contaminated food and water, inhalation of contaminated air or dust, and contact with consumer products or contaminated soil. Studies have shown that PFAS can accumulate in the environment and bioaccumulate in the food chain, leading to human exposure through consumption of contaminated seafood, meat, produce, and dairy products [1,2]. PFAS can also leach from food packaging materials, such as paper and board coatings, into food products, contributing to dietary exposure [3]. Occupational exposure to PFAS can occur among workers in industries involved in the production or use of PFAS-containing products, such as firefighters using firefighting foams containing PFAS [4]. Furthermore, exposure to PFAS can occur during the use of consumer products treated with PFAS, such as textiles, carpets, and non-stick cookware, through dermal contact and inhalation of airborne particles [5]. Overall, these exposure pathways highlight the need for comprehensive monitoring and regulation to mitigate human exposure to PFAS and protect public health.

Exposure to PFAS has been associated with a range of adverse chronic health effects. PFAS have been found to accumulate in and damage the liver tissue, causing oxidative stress, inflammation, hepatomegaly, fibrosis, and steatosis [6,7,8]. PFAS can lead to alterations in lipid profiles, including increased serum cholesterol and triglyceride levels, which are risk factors for fatty liver disease [9]. PFAS exposure has also been correlated with disruption of endocrine function, which in turn can lead to hormone imbalances, general changes in hormone levels, changes to hormone signaling pathways, reproductive abnormalities, and adverse reproductive outcomes [10,11,12,13]. PFAS have also been linked to increased occurrence of certain types of cancers, with definitive links to testicular and kidney cancer, along with possible links to prostate and breast cancer [11,14,15,16,17,18].

While blood is an important component in the understanding of a person’s exposure profile, studies have shown that whole blood, plasma, and serum matrices are inherently different in their composition of PFAS compounds and each tell a unique story of a person’s exposure to PFAS [19]. Generally, serum/plasma to whole blood is at a 2:1 ratio of PFAS concentration; however, all PFAS compounds do not have the same affinity to be found in the different blood matrices and that definitive ratio can vary [19,20,21,22]. While PFAS analysis in serum is most widely used, DBS are preferable in certain instances (children, remote/resource-limited settings, ease of shipping and storage, smaller sample volume requirement, trypanophobia) and is a crucial tool for studying PFAS exposure.

While many studies of PFAS in human blood focus solely on the serum component [23,24,25], far fewer methods are available for the study of PFAS in whole blood [20,21,22,26,27], and even fewer for the study of PFAS in DBS [28,29,30,31]. The current literature of PFAS in DBS methods have large sample sizes (whole spots or up to ten DBS punches), online solid phase or ion pair extractions, and majorly study only PFHxS, PFOA, and PFOS with reporting limits in the low ng mL^−1^ range [28,29,30,31]. The method developed and presented here features a simple extraction with a clean-up step, ng L^−1^ reporting limits, and an expansive panel of 39 PFAS compounds: (Table 1) perfluorocarboxylic acids (PFBA, PFPeA, PFHxA, PFHpA, PFOA (linear and branched), PFNA, PFDA, PFUnA, PFDoA, PFTriA, PFTeA), perfluorosulfonic acids (PFPrS, PFBS, PFPeS, PFHxS (linear and branched), PFHpS, PFOS (linear and branched), PFNS, PFDS, fluorotelomer sulfonic acids (4:2 FTS, 6:2 FTS, 8:2 FTS), perfluorosulfonamidoacetic acids (MeFOSAA, EtFOSAA), perfluorosulfonamides (PFBSA, PFHxSA, PFOSA), and a variety of other new/replacement PFAS (PFECHS, NFDHA, PFEESA, PFMPA, PFMBA, HFPO-DA, ADONA, 9Cl-PF3ONS, 11Cl-PF3OUdS). The high throughput of this method allows for the rapid and routine analysis of a small DBS sample size (two 6 mm punches) while maintaining low ng L^−1^ reporting limits.

This proven analytical method presented here has been used over the past three years by the State of Michigan Department of Health and Human Services (MDHHS) to quantify 39 PFAS in DBS for targeted biomonitoring studies throughout the state, including the Michigan PFAS Exposure and Health Study [32]. Since 2021, this method has been utilized to report PFAS concentrations in human DBS for over 2500 Michigan residents. This paper outlines the complexities of our method, detailing every essential phase from preparation of samples to separation (chromatographic) and detection (mass spectrometric). Additionally, we demonstrate the strength and dependability of our approach through comprehensive validation studies, which cover method accuracy, precision, sensitivity, linearity, and specificity.

## 2. Experimental

### 2.1. Chemicals and Reagents

All PFAS compounds covered by this method can be found in Table 1, which lists their names, abbreviations, and CAS numbers.

A stock solution of internal standards was prepared by utilizing the labeled internal standards outlined in Table 1 from Wellington Laboratories (Guelph, ON, Canada), achieving a concentration of 1.53 ng mL^−1^ in LC-MS grade methanol (Honeywell Burdick and Jackson^TM^, Muskegon, MI, USA). Analytes were quantitated using a corresponding labeled internal standard if a matching labeled internal standard was available (Table 1). Analytes were quantitated using a surrogate labeled internal standard which was determined to be a suitable match based on structure and/or retention time if a matching labeled internal standard was not available (Table 1).

External calibration standards and a continuing calibration verification (CCV) sample, each containing all PFAS analytes, were diluted in 1.25 mM sodium hydroxide in LC-MS grade methanol. Materials were prepared in-house taking into account matching of the volume of a 6 mm DBS (literature estimates 8.7 µL [33] per 6 mm DBS) to be used at the following concentrations at the analytical instrument (concentrations listed apply to all analytes unless noted otherwise due to some of the analytes only being available as mixtures): 0.05 ng mL^−1^ (0.04055 ng mL^−1^ L-PFHxS, 0.00945 ng mL^−1^ B-PFHxS), 0.1 ng mL^−1^ (0.0811 ng mL^−1^ L-PFHxS, 0.0189 ng mL^−1^ B-PFHxS), 0.5 ng mL^−1^ (0.4055 ng mL^−1^ L-PFHxS, 0.0945 ng mL^−1^ B-PFHxS), 1.0 ng mL^−1^ (0.811 ng mL^−1^ L-PFHxS, 0.189 ng mL^−1^ B-PFHxS), 5.0 ng mL^−1^ (4.055 ng mL^−1^ L-PFHxS, 0.945 ng mL^−1^ B-PFHxS), 10 ng mL^−1^ (8.11 ng mL^−1^ L-PFHxS, 1.89 ng mL^−1^ B-PFHxS), 50 ng mL^−1^ (40.55 ng mL^−1^ L-PFHxS, 9.45 ng mL^−1^ B-PFHxS), CCV 0.25 ng mL^−1^ (0.203 ng mL^−1^ L-PFHxS, 0.047 ng mL^−1^ B-PFHxS). All analyte spike materials were purchased from Wellington Laboratories (Guelph, ON, Canada) (Table 1). These materials were used for calibration curves for all experiments that follow, as well as the reportable range verification study, and analytical specificity.

Matrix-matched quality control (QC) materials containing all PFAS analytes were diluted into pre-screened bovine blood (heparin, Sierra for Medical Science, Whittier, CA, USA) in-house and 50 µL was spotted onto a Whatman^TM^ 903 Proteinsaver sample collection card (Cytiva, Marlborough, MA, USA) at the following concentrations (concentrations listed apply to all analytes unless noted otherwise due to some of the analytes only being available as mixtures): 0.40 ng mL^−1^ (0.324 ng mL^−1^ L-PFHxS, 0.076 ng mL^−1^ B-PFHxS), 1.5 ng mL^−1^ (1.215 ng mL^−1^ L-PFHxS, 0.285 ng mL^−1^ B-PFHxS), 15 ng mL^−1^ (12.15 ng mL^−1^ L-PFHxS, 2.85 ng mL^−1^ B-PFHxS). Standards for each PFAS analyte were purchased from Wellington Laboratories (Guelph, ON, Canada) as detailed in Table 1. Although bovine blood is not a perfect match for human blood, bovine blood was found to contain significantly lower background PFAS contamination than human blood, which was crucial to the study considering the aim to spike PFAS materials into the blood for matrix-matched quality control materials. Heparinized bovine blood was screened for PFAS contamination prior to use and the level of PFAS contamination was characterized and resulted in higher-than-spiked QC material levels in some cases. Although sodium heparin bovine blood was utilized, lithium heparin is also acceptable but use of EDTA should be avoided if possible [34]. Hematocrit was not measured or normalized. These matrix-matched QC materials were used to ensure QC throughout all the subsequent experiments and served as the test material for the study of accuracy/recovery and precision.

Nine additional levels of materials were prepared for additional studies of accuracy, precision, and sensitivity, as well as served as internal proficiency testing samples. These materials each contained all PFAS analytes diluted into pre-screened bovine blood in-house and 50 µL was spotted onto a Whatman^TM^ 903 Proteinsaver (Cytiva, Marlborough, MA, USA) sample collection card at the following concentrations (concentrations listed apply to all analytes unless noted otherwise due to some of the analytes only being available as mixtures: 0 ng mL^−1^, 0.005 ng mL^−1^ (0.0041 ng mL^−1^ L-PFHxS, 0.00095 ng mL^−1^ B-PFHxS), 0.010 ng mL^−1^ (0.0081 ng mL^−1^ L-PFHxS, 0.0019 ng mL^−1^ B-PFHxS), 0.020 ng mL^−1^ (0.0162 ng mL^−1^ L-PFHxS, 0.0038 ng mL^−1^ B-PFHxS). 0.040 ng mL^−1^ (0.0324 ng mL^−1^ L-PFHxS, 0.0076 ng mL^−1^ B-PFHxS). 0.120 ng mL^−1^ (0.0972 ng mL^−1^ L-PFHxS, 0.0228 ng mL^−1^ B-PFHxS). 0.400 ng mL^−1^ (0.324 ng mL^−1^ L-PFHxS, 0.076 ng mL^−1^ B-PFHxS). 4.0 ng mL^−1^ (3.24 ng mL^−1^ L-PFHxS, 0.76 ng mL^−1^ B-PFHxS). 40.0 ng mL^−1^ (32.4 ng mL^−1^ L-PFHxS, 7.60 ng mL^−1^ B-PFHxS). Standard materials for each PFAS analyte were purchased from Wellington Laboratories (Guelph, ON, Canada) as detailed in Table 1. Heparinized bovine blood was screened for PFAS contamination prior to use. These matrix-matched materials were used as the test material for the study of accuracy, precision, and sensitivity/limit of detection. In addition, the 40.0 ng mL^−1^ sample was utilized to study carryover, maximum dilution, and preferred dilution scheme.

Taurodeoxycholic acid (TDCA, EMD Millipore©, Burlington, MA, USA) was prepared at several concentrations above biological relevance [26,35] (4.983 mg L^−1^, 49.83 mg L^−1^) and mixed with medium- (1.5 ng mL^−1^) level matrix-matched QC material to study a potential cross-reactivity and interferences with PFAS compounds.

LC-MS grade water, LC-MS grade methanol, LC-MS grade acetonitrile, and LC-MS grade ammonium acetate were purchased from Honeywell Burdick and Jackson (Muskegon, MI, USA).

### 2.2. Three-Dimensional Printed Labware Fabrication

To minimize potential contamination and analyst error, the number of handling steps for the DBS specimens was reduced by designing a custom 3D-printed 24-well plate that accommodated 2.0 mL microcentrifuge tubes used for extraction. By designing the 3D-printed custom labware to mirror the layout a 24-well plate, the final product was amenable to an automated DBS puncher, eliminating the need to manually transfer DBS from a collection plate to the microcentrifuge tube for the extraction step. Instead, the custom labware allows the DBS to be punched directly into the extraction microcentrifuge tubes.

**Figure 1:**
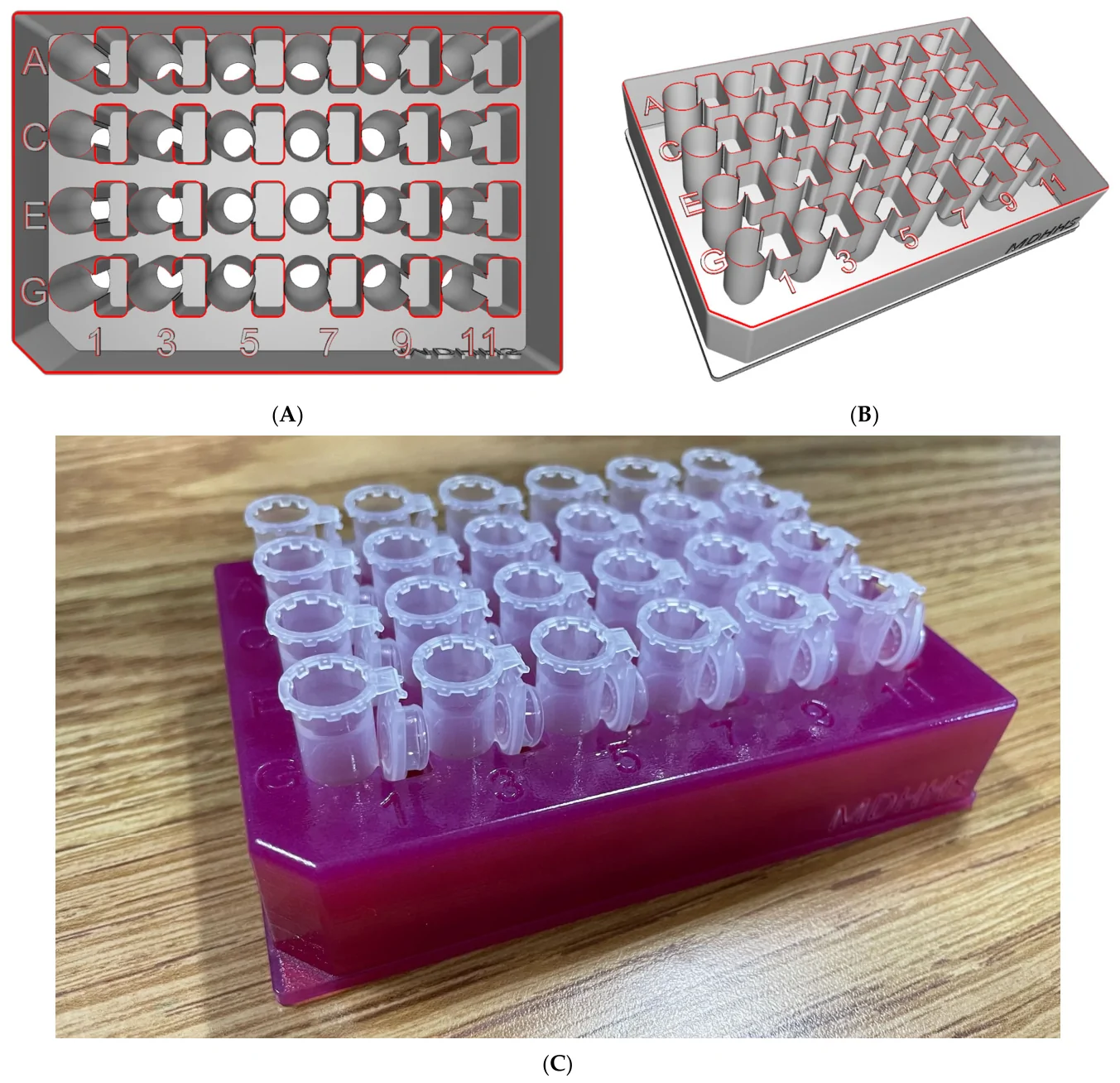
3D-Printed Labware as visualized from two different angles (**A**,**B**) and with 2.0 mL microcentrifuge tubes (**C**).

The .stl files for the 3D-print design were designed using Autodesk^TM^ Inventor Professional software (Build 158, Version 27.0.15800.0000, Autodesk, San Francisco, CA, USA). The 3D-printed custom labware was fabricated using Vero^®^ materials on a Stratasys J735™ PolyJet printer. A visualization of the 3D-printed DBS puncher plate can be seen in Figure 1.

### 2.3. Sample Preparation

**Figure 2:**
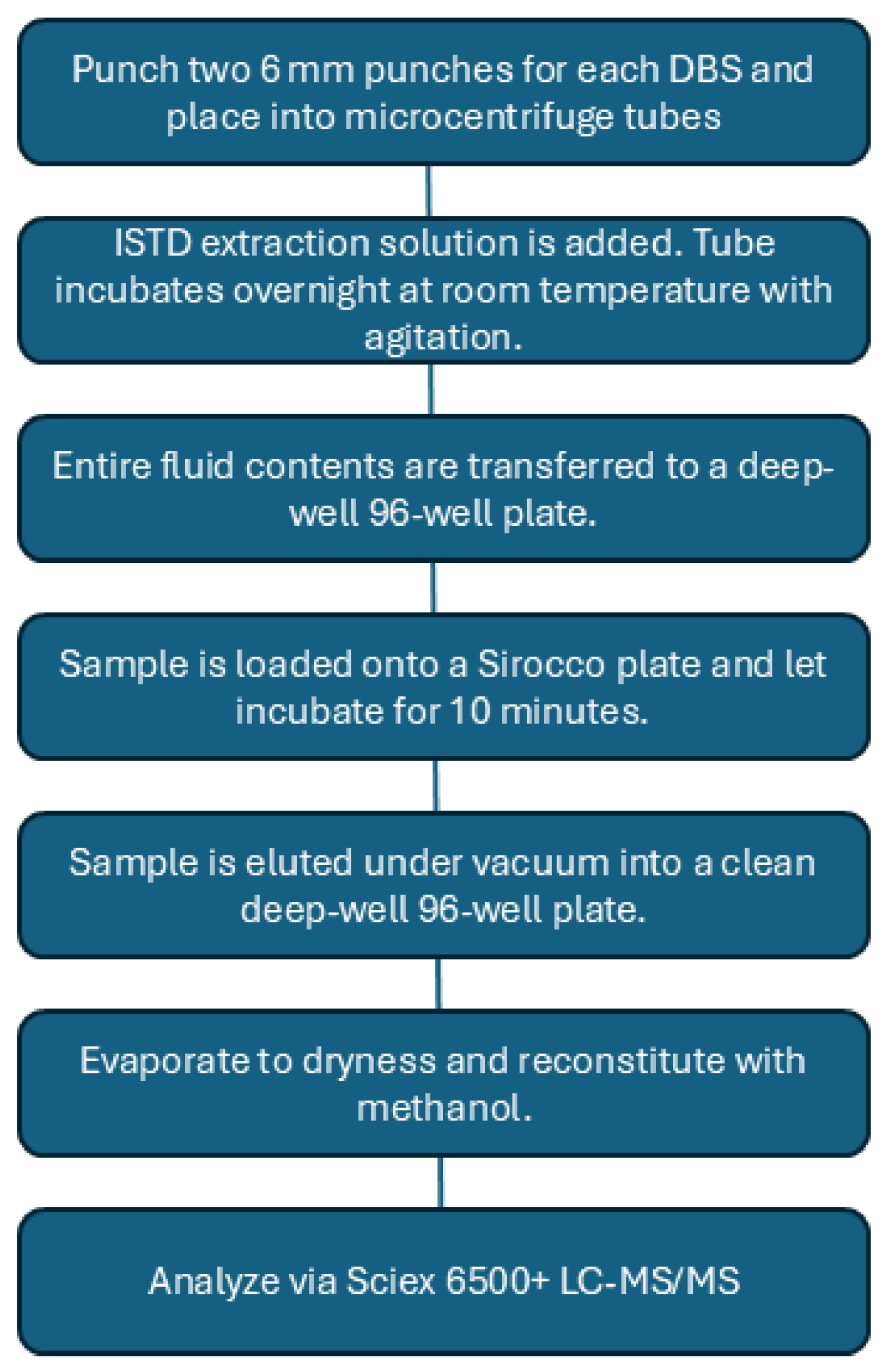
PFAS DBS Workflow.

Figure 2 shows a schematic of the PFAS DBS sample preparation workflow. Two 6 mm DBS were punched using a methanol-cleaned punch head on a DBS puncher (Revvity©, Waltham, MA, USA) directly into a 2.0 mL microcentrifuge tube (Eppendorf Safe-Lock, Enfield, CT, USA) using the custom 3D-printed microcentrifuge tube holder described in Section 2.2 (Figure 1). On a DBS card, punches were taken from the center of two distinct blood spots if possible. In addition, punches were taken from the leftmost and rightmost acceptable spots on a card. As procurement of PFAS-free DBS cards was not found to be possible, at least five method blanks were punched for each batch of samples, ideally from the same lot of DBS cards as the samples were spotted on. While subtraction of the background PFAS levels in DBS cards in this study was not completed, background subtraction from an average of the PFAS concentrations in the five method blanks would be performed if patient samples were to be analyzed. In addition, blank DBS cards were also punched and extracted for the external calibration curve and CCVs in an effort to correct for inherent blank DBS card PFAS contamination, as the blank DBS card material lot was consistent between all material prepared for this study.

To each microcentrifuge tube containing DBS samples and method blanks, 1.5 mL of extraction solution (LC-MS grade methanol containing 0.051 ng mL^−1^ internal standard mixture) is added to the tube. To each microcentrifuge tube containing blank filter paper for the calibration curve and CCVs, 1.5 mL of LC-MS grade methanol is added. This creates a novel and hybrid matrix-matched solvent-based calibration curve. Tubes are vortexed overnight at 700 rotations per minute (rpm) on a VWR DVX-2500 multi-tube vortexer (VWR^TM^, Radnor, PA, USA). Following the overnight extraction, the total liquid contents of each microfuge tube are transferred to a TrueTaper^®^ 96-well deep-well plate (Analytical Sales and Services©, Flanders, NJ, USA).

The 96-well sample plate on a Tomtec liquid handler (Tomtec© Quadra 3 and Quadra 4, Hamden, CT, USA) was utilized. The total contents of the TrueTaper 96-well deep-well plate were transferred to a Waters Sirocco™ Protein Precipitation plate (Waters, Milford, MA, USA) in two loading steps, each with a 10 min incubation period on the Sirocco™ plate prior to elution under vacuum into a clean TrueTaper^®^ 96-well deep-well plate.

The samples were taken to dryness under a stream of nitrogen using an SPE Dry (Biotage, Uppsala, Sweden) with temperature settings of 60 °C upper and 80 °C lower, and a flow setting of 40 L min^−1^ nitrogen. The sample plate was allowed to cool to room temperature prior to reconstitution. The DBS samples and method blanks were reconstituted using 100 µL of LC-MS grade methanol. The DBS calibration curve and CCV blanks were reconstituted with the respective external calibration and CCV mixtures (50 µL) as prepared in 2.1 and 50 µL of an internal standard stock solution. The VWR multi-tube vortexer was utilized for sample mixing following the placement of a silicone cap mat (Analytical Sales and Services).

### 2.4. LC-MS/MS Analysis

Two identical Sciex 6500+ mass spectrometers coupled with Sciex Exion LC systems (Sciex, Framingham, MA, USA) were utilized for all assays that follow. These high-performance triple quadrupole mass spectrometers operated in MS/MS multiple reaction monitoring (MRM) mode with negative ion detection using an electrospray ionization (ESI) source. The LC-MS/MS system was configured and data were acquired using the Analyst software version 1.7.1, and data processing was carried out with Sciex OS-MQ software version 2.2.

The column oven was held at a temperature of 40 °C. A Restek Raptor C18 column (2.1 × 150 mm, 2.7 µm, Bellefonte, PA, USA) was used as an analytical column and a Restek Raptor C18 column (2.1 × 50 mm, 2.7 µm) was used as a delay column located between the mixer and the autosampler as a way to delay PFAS contamination from the LC system. The LC utilized a binary gradient pumping mode with a gradient of 2 mM ammonium acetate in LC-MS grade water (mobile phase A, MPA) and LC-MS grade acetonitrile (mobile phase B, MPB) at 0.3 mL min^−1^. Initially, the ratio of MPB was 5%, which was maintained for 2.0 min, then increased to 50% MPB over 6.0 min, then increased to 85% MPB over 5.0 min, and finally reached 100% MPB at 14.0 min. The gradient then reverted to 5% MPB for 1.0 min, resulting in a total run time of 15.0 min. The injection volume was 4 μL. The column dead volume was 0.15 mL. With a calibration curve, blanks, and quality control materials, approximately 70 DBS samples can be analyzed per day.

The Sciex 6500+ MS was operated with the following parameters: curtain gas—20 L min^−1^, interface temperature—300 °C, ion source gas 1–30 L min^−1^, ion source gas 2–40 L min^−1^, collision gas—12 L min^−1^, IonSpray voltage—2000 V. Voltages (declustering potential, entrance potential, collision energy, and collision exit potential) for each analyte ion measured can be found in Morrison et al. [36].

**Figure 3:**
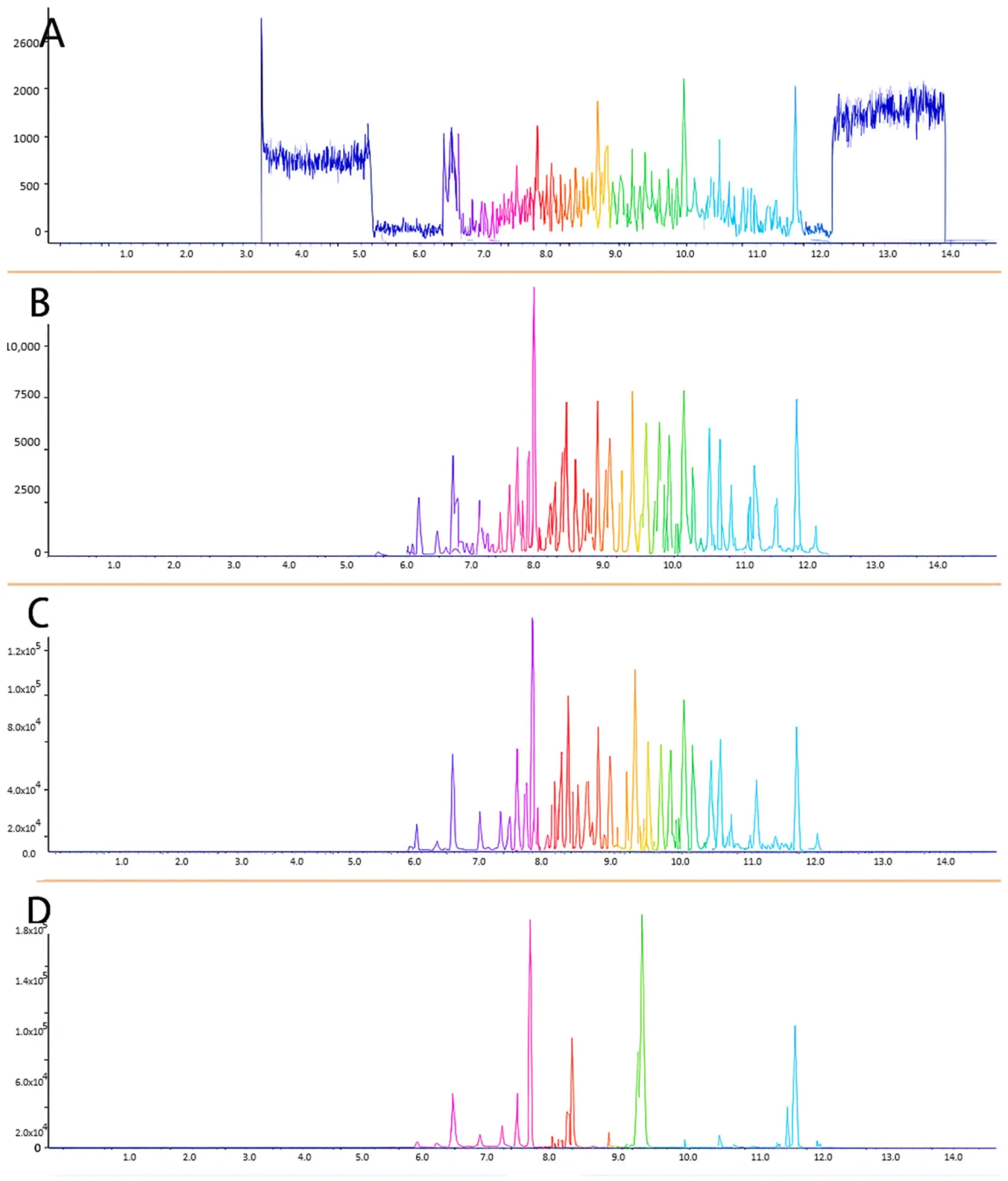
Representative Sciex 6500+ Chromatograms. (**A**) Blank DBS. (**B**) Lowest calibrator (50 ng L^−1^). (**C**) Quality control sample. (**D**) Representative human sample.

Please see Figure 3 for representative chromatograms. Figure 3A represents a blank DBS sample, Figure 3B represents the lowest calibrator (50 ng L^−1^), Figure 3C represents a typical quality control sample, and Figure 3D is representative of a human sample.

### 2.5. Method Validation

For all validation studies that follow, CLSI validation guidelines were followed for CAP accreditation. Data collected was included in the study if it met the appropriate criteria for calibration curve regression (R^2^ ≥ 0.9900), calibration curve point accuracy (≥70%), peak signal-to-noise ratio (≥10:1 for the ion used for quantification and ≥3:1 for the ion used for confirmation), and confirmatory ion signal ratios (run-specific ratio, calculated as the averaged confirmatory ratio across all calibrators ± 30%). Calibration curves were linear fit and 1/x weighting was utilized.

For PFHxS, PFOA, and PFOS, stock materials containing both linear and branched compounds were utilized. For these analytes, results for the “L-” represent the linear isomer analyte quantitated using the integration of linear isomer peaks only. Results for the “B-” represent the branched isomer analyte quantitated using the integration of branched isomer peaks only. Total-PFHxS, -PFOA, -PFOS (sum of branched and linear isomers) are not reported in this study.

#### 2.5.1. Accuracy

Samples used for the assessment of accuracy were in-house-prepared internal proficiency testing samples and QC materials prepared as described in Section 2.1. Samples were evaluated through separate, duplicate extractions and analyzed daily over several days by multiple scientists using two identical LC-MS/MS systems. The laboratory’s established performance criterion for accuracy was set at ≥70%. Samples were evaluated against the target value for these samples. Due to the use of an external calibration curve in the solvent, method recovery is being treated as the same as accuracy for the purposes of this study.

#### 2.5.2. Precision

Samples used to assess precision were in-house-prepared internal proficiency testing samples and QC materials prepared as described in Section 2.1. Samples were evaluated through separate, duplicate extractions and analyzed each day over multiple days by several scientists using two identical LC-MS/MS systems. Inter-day, intra-day, and overall method precision were examined. For each level, the mean and standard deviation were calculated, and the total coefficient of variation (CV%) was determined. Additional variance components were calculated to assess intra-day and inter-day precision. The laboratory’s performance criterion for total precision was defined as ≤30% imprecision.

#### 2.5.3. Reportable Range

Accuracy across the analytical measurement range was confirmed by quantifying calibration standards against a calibration curve with a coefficient of determination (R^2^) of at least 0.9900, ensuring reliable performance throughout the measurement range. Two independent calibration curves were generated by the dilution of calibration curve stock materials into LC-MS-grade methanol as described in Section 2.3. Calibration curves were assessed with regression analysis for the fit of their weighted least squares correlation. A residual analysis of the data was also generated and evaluated based on a systematic basis to understand the appropriateness of curve fit. The lowest calibration points were evaluated to ensure that it could be quantified using both signal-to-noise ratios (i.e., ≥10:1 for the quantification ion and ≥3:1 for the confirmation ion) and confirmatory ion signal ratios (run-specific ratio, calculated as the averaged confirmatory ratio across all calibrators ± 30%). The laboratory established a performance threshold of ≥70% accuracy for each calibration curve point to ensure reliability across the reportable range. Accuracy across this range was verified on both of the duplicate LC-MS/MS systems.

#### 2.5.4. Sensitivity, Limit of Detection

The Limit of Detection (LOD) and Limit of Quantitation (LOQ) were determined using a LINEST regression of the calculated concentrations for the three lowest calibrators from all calibration curves generated in the study. The standard deviation of the y-intercept divided by the slope of the least-squares regression line was used to calculate S_0_. The analytical LOD was defined as three times S_0_, and the analytical LOQ as ten times S_0_. The LOQ was required to fall below the lowest calibrator; otherwise, the assay had to be revised. Only calibration curves with an R^2^ value ≥ 0.9900 were included. Sensitivity was further evaluated by examining signal-to-noise ratios at the method’s reporting limits. The sensitivity and LOD assessment was conducted on both duplicate LC-MS/MS systems, with the laboratory’s criterion specifying that the LOQ must be less than or equal to the lowest calibration point.

#### 2.5.5. Carryover

Carryover was assessed for this method for three different scenarios: carryover occurring at the DBS puncher, during sample preparation, and at the LC-MS/MS. For carryover occurring during DBS punching, blank DBS punches following the punching of a 40.0 ng mL^−1^ sample were analyzed. If no analytes are detected in this sample above inherent PFAS levels, carryover on the DBS puncher is not observed. For evaluating potential carryover during sample preparation, the TomTec liquid handler was examined. Pipette tips were replaced after the sample transfer step, and LC-MS-grade methanol was dispensed with new tips into a clean 96-well plate. The plate was then dried, reconstituted, and analyzed. If no analytes are detected in this plate, carryover is not present in the system. For instrument carryover, the highest calibration point was analyzed before a double blank (no analyte, no internal standard) sample. This was assessed on each of two duplicate LC-MS/MS systems. No level of carryover would be deemed acceptable; if carryover were found to occur, remedial action would need to be performed to ensure sample contamination does not occur.

#### 2.5.6. Analytical Specificity

Guidance from The Best Practices (6.7.2.1) of the Clinical Laboratory Standards Institute (CLSI) LC-MS guidance document C62-A30 was utilized to plan and develop a measurement of analytical specificity [37]. This document states that results of <15% does not signify that matrix effects are significantly contributing to assay imprecision [36]. However, a result >15% does suggest that matrix effects are significantly contributing to assay imprecision [36]. Eight calibration curves were prepared in the solvent to be analyzed and a mean signal (peak area) was obtained from each calibration point for each analyte and each internal standard used. An additional eight calibration curves were prepared by spiking post-extraction from different lots of matrixes (bovine blood from different individuals). A mean signal (peak area) was obtained for each calibration point for each analyte and internal standard. Double blanks, blank filter paper, and matrix blanks were also run with each calibration curve. The mean signal from the blank filter paper was used to correct the mean signal for each calibration point prepared in solvent and the matrix blank was used to correct the mean signal for each calibration point prepared in the matrix. The blank corrected mean signal for the matrix-based calibration curves was compared to the blank corrected mean signal for the solvent-based calibration curves to determine matrix effects, which was calculated as a percent difference. A mean matrix effect for each analyte was calculated across all calibration curves. A normalized percent matrix bias was calculated by using the mean matrix effect of each internal standard to correct for the mean matrix effect of each analyte. As stated above, a normalized percent matrix bias of ≤15% was preferred.

#### 2.5.7. Interferences and Cross Reactivity

To evaluate potential interference from additional persistent organic pollutants (POPs) in PFAS DBS analysis, samples containing high concentrations of various POPs were prepared and compared with matrix-matched QC materials at the same concentrations. An average percent difference was calculated to estimate the level of POP-related interference.

To assess possible interference from bile acids, TDCA was prepared at biologically relevant concentrations (4.983 mg L^−1^, 49.83 mg L^−1^) [26,35] and mixed with the medium-level matrix-matched QC material to examine chromatographic and analytical effects on PFAS quantitation. Interference from TDCA was estimated by calculating the average percent difference relative to the medium-level QC material without TDCA.

#### 2.5.8. Maximum Dilution

An experiment to test the maximum dilution and dilution scheme for DBS was performed utilizing the 40.0 ng mL^−1^ sample prepared in Section 2.1. Two different types of dilutions were performed: (1) pre-extraction using smaller or fewer DBS or (2) post-extraction using a diluent that contained labeled internal standard. For the pre-extraction study, six individually extracted replicates were performed at each of the following dilutions: 1:2, 1:4, and 1:8. Dilutions were made possible by using fewer or smaller (3 mm) spots. For this study, extraction occurred as detailed in Section 2.3, but with a smaller sample size as the dilution. For the post-extraction study, six individually extracted replicates were diluted post-extraction by dilution in an internal standard dilution solution to match the labeled internal standard concentration following the procedure. Dilutions of 1:2, 1:4, and 1:8 were performed to match and compare to the pre-extraction study.

## 3. Results and Discussion

### 3.1. Accuracy

Accuracy describes how closely a measurement, or the average of repeated measurements, matches a true or reference value. Bias, which quantifies accuracy, is defined in CLSI EP15-A2 [38] and International Organization for Standardization (ISO) 3534-1 [39] as the difference between the expected test result and the accepted reference value. Accuracy is also referred to as trueness. For this method, the laboratory’s acceptance criterion required all measurements to fall within 30% of the expected value (70–130%) to be considered accurate. For the purposes of this study, due to the use of an external solvent-based calibration curve and inherent levels of PFAS contamination present in clean DBS cards, accuracy results are being treated as synonymous with recovery results.

Laboratory-generated samples were prepared using materials independent of the calibrator to evaluate accuracy. For each analyte, values from all levels across all batches were compared with the expected targets. The experimental averages for the internal proficiency-testing samples were within an acceptable ≥81.3% of the target value for all analytes, and the QC materials met an acceptable ≥81.8% of the target value for all analytes (Table 2).

Through initial work with the Whatman^TM^ 903 Protein Saver cards it was noted that there was significant and variable PFAS contamination present inherently on these cards. Due to this unavoidable certainty, a blank subtraction was utilized in an effort to adequately capture native DBS card PFAS contamination that may affect sample results. For the purposes of this method, five method blanks taken from across a blank DBS card were analyzed and averaged to subtract from analysis results. Through accuracy studies, PFHxA, PFHpA, PFPeA, and 6:2 FTS showed the most consistent DBS card contamination, which results in these compounds having an increased reporting limit for this method to account for the need to background subtract for these compounds, and the inherent variability in doing so. These four compounds will have a 200 ng L^−1^ reporting limit for this method. Note that for PFHxA, PFHpA, PFPeA, and 6:2 FTS, the highly variable lowest level tested (60 ng L^−1^) was not included in the average calculation due to the inherent background contamination in the DBS cards and the increased reporting limit for these compounds. All accuracies hit the desired ≥70% for both internal proficiency testing samples and QC samples.

### 3.2. Precision

According to CLSI EP05-A2 [40] and ISO 3534-1 [39], precision is defined as the degree of agreement among independent measurements made under specified conditions. This method evaluates the total within-laboratory precision for the procedure, including variance associated with intra-day and inter-day factors. In this study, precision results were expressed as imprecision, with a target of ≤30%. Each laboratory-generated sample was extracted individually in at least duplicate either within a single run (intra-day precision) or across multiple days (inter-day precision). All analytes demonstrated <27.4% imprecision for both intra-day and inter-day assessments, with most showing <20% imprecision (Table 2).

### 3.3. Reportable Range

The accuracy of the analytical measurement range was confirmed by treating individual calibration curve points as unknowns and quantifying them against a validated calibration curve. The accuracy of each point was then averaged to determine overall accuracy across the reportable range. A ±30% threshold served as the acceptance criterion for calibration-point accuracy during each batch run. Table 3 presents the accuracy results for the reportable range, with accuracy values for each analyte falling between 70.9% and 100%, demonstrating excellent performance across the range.

### 3.4. Sensitivity, Limit of Detection

The LOD and LOQ were determined using the LINEST regression method. Only calibration curves with R^2^ ≥ 0.9900 and acceptable signal-to-noise ratios (≥10:1 for the quantifier ion and ≥3:1 for the qualifier ion) were included. The target criterion required the LOQ ≤ the lowest calibrator (S1), allowing S1 to serve as the assay’s reporting limit. For all analytes, the LOQ met this requirement (Table 3), and S1 was therefore used as the reporting limit (50 ng L^−1^ for all analytes, except L-PFHxS (41 ng L^−1^), B-PFHxS (9 ng L^−1^), PFPeA (200 ng L^−1^), PFHxA (200 ng L^−1^), PFHpA (200 ng L^−1^), and 6:2 FTS (200 ng L^−1^). Reporting limits were set higher for PFPeA, PFHxA, PFHpA, and 6:2 FTS as these analytes were found to have the most frequent inherent DBS card contamination.

### 3.5. Carryover

Carryover refers to PFAS remaining in the system and transferring into subsequent samples after high-concentration or over-range samples are analyzed. Assessing carryover helps determine whether results following such samples can be considered reliable. Carryover was evaluated at the DBS puncher and the TomTec liquid handlers. No analyte peaks were detected, indicating no carryover at the puncher or in the solvent dispensed after the TomTec handled high-concentration materials. Carryover at the LC-MS/MS was assessed by analyzing blank injections following the highest calibrator and over-range samples. No analyte peaks were observed in these blanks on either instrument platform.

### 3.6. Analytical Specificity

Specificity investigates whether matrix effects contribute to assay imprecision. The Best Practices (6.7.2.1) of the CLSI LC-MS guidance document C62-A30 [37] states that results of <15% does not signify that matrix effects are significantly contributing to assay imprecision. To test this, calibration curves were generated by preparing calibrator stock solutions to be used for spiking into a variety of whole blood matrices and spotted onto DBS cards. Eight stock calibrator spiking solutions of increasing concentration were diluted into whole blood matrix and spotted onto DBS cards. Eight calibration curves in the matrix were analyzed and a mean signal (peak area) was obtained from each calibration point for each analyte and internal standard used. A calibration curve was generated by preparing calibrators in methanol at the concentrations expected following the assay. Eight solvent-based calibration curves were analyzed, and a mean signal (peak area) was obtained from each calibration curve point for each analyte and internal standard used. Double blanks, blank filter paper, and matrix blanks were also run with each calibration curve. The mean signal from the blank filter paper was used to correct the mean signal for each calibration point prepared in the solvent and the matrix blank was used to correct the mean signal for each calibration point prepared in matrix. The blank corrected mean signal for the matrix-based calibration curves was compared to the blank corrected mean signal for the solvent-based calibration curves to determine matrix effects, which was calculated as a percent difference. A mean matrix effect for each analyte was calculated across all calibration curves. A normalized percent matrix bias was calculated by using the mean matrix effect of each internal standard to correct for the mean matrix effect of each analyte. All results were <11% (Table 3), demonstrating that matrix effects are not contributing to assay imprecision. Additional calculations on matrix effects, including matrix factor, internal standard matrix factor, internal standard normalized matrix factor, and a raw coefficient of variation in peak areas over the eight different matrix lots can be found in Table S1. These additional calculations also demonstrate that matrix effects are minimal and comparable across eight different sources of bovine serum.

Throughout the development of this analytical method, extensive steps were taken to reduce inaccuracies caused by interfering signals, including those from endogenous or environmental chemical sources, degradation products formed during handling or storage, impurities in internal standards, or isomers present in the sample. Specificity in this LC-MS/MS method is achieved through multiple approaches. Sample preparation incorporates protein precipitation and filtration to remove chemical interferents, while LC separation—supported by the use of a delay column—effectively resolves analytes from potential interfering compounds. MS further enhances specificity by utilizing selective ion fragmentation pathways to eliminate co-occurring chemical interference. Additionally, isotopically labeled internal standards are employed to reduce matrix effects. However, in cases where appropriate isotopically labeled standards are unavailable, matrix suppression may still pose a challenge.

### 3.7. Interferences and Cross Reactivity

To evaluate the impact of additional POP interference on PFAS DBS analysis, samples were prepared with high concentrations of various POPs and compared with matrix-matched QC materials at the same levels. An average percent difference was calculated to estimate the degree of POP-related interference.

Chromatographic interferences caused by high concentrations of POPs were evaluated by spiking and spotting blood with high levels of POPs and comparing the recovery back to a control without POPs added. See Table 4 for method interference results. Between the two POP concentrations tested, all analytes showed a ≤19% difference from the control. It should be noted that none of the POP-containing samples had chromatographically interfering peaks.

To evaluate potential interference from bile acids, TDCA was prepared at biologically relevant concentrations (4.983 mg L^−1^, 49.83 mg L^−1^) [26,35] and combined with the medium-level matrix-matched QC material to assess chromatographic and analytical effects on PFAS quantitation. Interference from TDCA was estimated by calculating the average percent difference relative to the medium-level QC material without TDCA. See Table 5 for method interference results. Between the two biologically relevant TDCA concentrations, all analytes showed a ≤22% difference from the control. It should be noted that none of the TDCA-containing samples had chromatographically interfering peaks with the PFAS compounds of interest.

### 3.8. Maximum Dilution

The reportable range may be extended beyond the reportable range if over-range or high samples can accurately be diluted to within the calibration range. This is called the maximum dilution. The maximum dilution experiment consisted of six individually extracted replicates at 1:2, 1:4, and 1:8 dilutions and was analyzed using two different dilution schemes. The two dilution schemes are as follows: (1) pre-extraction using a smaller or fewer DBS or (2) post-extraction using a diluent that contained ISTD. These dilution schemes were compared to determine the most accurate means of diluting high samples.

The average calculated concentration across six individually extracted replicates was calculated for each parameter (undiluted, pre-extraction dilution (1:2, 1:4, 1:8), and post-extraction dilution (1:2, 1:4, 1:8)). Each dilution was compared to the expected concentration by using a percent difference calculation (Table 5). The percent difference was then compared across all dilutions. Based on internal standard recovery, it was determined that a “correction factor” needed to be employed to account for internal standard loss over the extraction procedure. Data corrected with this ISTD “correction factor” can also be seen in Table 5.

The data shows that the preferrable scheme for dilution will be the pre-extraction scheme due to the inherent ability to account for analyte loss during the extraction by smaller or fewer DBS. Due to limitations on the quantity and size of DBS punches available, the maximum dilution that can be used is a 1:8 dilution.

## 4. Conclusions

This method has certain limitations that should be noted. Background interference can arise from the DBS cards used, as well as from the blank whole blood utilized in preparing the QC materials. Due to the inability to source blood free of PFAS compounds, calibrators could not be prepared directly in the matrix. Instead, a filter paper punch was extracted and then spiked with calibrator in the extract prior to analysis to create a novel hybrid matrix-match and solvent-based calibration curve. This approach ensures that background interference from the filter paper is accounted for during calibration and reflected when analyzing samples. However, while the same lot of filter paper is used for the calibration curve punch and QC materials, it may differ from the lot used for the samples. Since a PFAS-free blood source could not be identified, bovine blood was selected to be used in the preparation of quality control materials. Although the bovine blood utilized did contain PFAS contamination, it was significantly less than the PFAS contamination seen in the human blood sources that were tested. It is recognized that bovine blood is not a direct equivalent to human blood, as bovine and human blood differ in protein binding, cellular composition, and potential matrix effects; however, we determined the benefits of having matrix-matched quality control materials spiked at low concentrations to outweigh the limitations of not utilizing human blood. In addition, hematocrit was not measured or normalized and may affect the spreading of blood across a DBS card, the volume of blood within a punch, and the efficiency of extraction. An additional limitation of the method lies in the unavailability of certified reference materials in the matrix which necessitates that study samples consist entirely of laboratory contrived materials, though efforts were made to utilize different lots or vendors when possible. A final limitation is the use of LINEST calculations to calculate the method LOQ in lieu of an experimental approach.

This manuscript presents a novel LC-MS/MS method for quantifying 39 PFAS analytes in DBS samples. The method incorporates a hybrid matrix external calibration curve, requires minimal sample volume, and uses a streamlined preparation process with analyte concentration to achieve low-ng L^−1^ LODs. It meets or exceeds targeted criteria for accuracy/recovery, precision, sensitivity, carryover, specificity, and verification of the reportable range. Over the past three years, this validated method has been essential for generating biomonitoring data for Michigan residents. In addition to the data shown, quality control sample results over time have a <5% failure rate, and triannual routine proficiency testing has resulted in a <1% failure rate. Further longitudinal stability studies are in progress. It holds strong potential for enhancing our understanding of PFAS exposure patterns in Michigan and beyond, supporting more targeted regulatory decisions and public health actions. As this method continues to evolve and be applied in epidemiological and environmental monitoring efforts, it will contribute to informed decision-making and proactive strategies aimed at reducing the effects of PFAS contamination on both human health and the environment.

## Figures and Tables

**Table 1:** PFAS analyte material sources with associated abbreviations, vendor, and CAS number (#). All materials available through Wellington Laboratories (Guelph, ON, Canada).

Abbreviated Name	Name	CAS#	Calibrator Material	Quality Control Material	Internal Standard Utilized
PFBA	Perfluorobutanoic acid	375-22-4	PFBA, 50 mg L^−1^	PFC-MXA, 2 mg L^−1^	^13^C-PFBA
PFPeA	Perfluoropentanoic acid	2706-90-3	PFPeA, 50 mg L^−1^	PFC-MXA, 2 mg L^−1^	^13^C-PFPeA
PFHxA	Perfluorohexanoic acid	307-24-4	PFHxA, 50 mg L^−1^	PFC-MXA, 2 mg L^−1^	^13^C-PFHxA
PFHpA	Perfluoroheptanoic acid	375-85-9	PFHpA, 50 mg L^−1^	PFC-MXA, 2 mg L^−1^	^13^C-PFHpA
L-PFOA	Perfluorooctanoic acid	335-67-1	PFOA, 50 mg L^−1^	PFC-MXA, 2 mg L^−1^	^13^C-PFOA
B-PFOA	Perfluoro-5-methylheptanoic acid		P5MHpS, 2 mg L^−1^	P5MHpS, 2 mg L^−1^	^13^C-PFOA
PFNA	Perfluorononanoic acid	375-95-1	PFNA, 50 mg L^−1^	PFC-MXA, 2 mg L^−1^	^13^C-PFNA
PFDA	Perfluorodecanoic acid	335-76-2	PFDA, 50 mg L^−1^	PFC-MXA, 2 mg L^−1^	^13^C-PFDA
PFUnA	Perfluoroundecanoic acid	2058-94-8	PFUdA, 50 mg L^−1^	PFC-MXA, 2 mg L^−1^	^13^C-PFUnA
PFDoA	Perfluorododecanoic acid	307-55-1	PFDoA, 50 mg L^−1^	PFC-MXA, 2 mg L^−1^	^13^C-PFDoA
PFTriA	Perfluorotridecanoic acid	72629-94-8	PFTrDA, 50 mg L^−1^	PFC-MXA, 2 mg L^−1^	^13^C-PFDoA
PFTeA	Perfluorotetradecanoic acid	376-06-7	PFTeDA, 50 mg L^−1^	PFC-MXA, 2 mg L^−1^	^13^C-PFTeA
PFPrS	Sodium Perfluoropropanesulfonic acid	423-41-6	PFPrS, 50 mg L^−1^	PFPrS, 50 mg L^−1^	^13^C-PFBS
PFBS	Perfluorobutanesulfonic acid	375-73-5	L-PFBS, 50 mg L^−1^	L-PFBS, 50 mg L^−1^	^13^C-PFBS
PFPeS	Perfluoropentanesulfonic acid	2706-91-4	L-PFPeS, 50 mg L^−1^	L-PFPeS, 50 mg L^−1^	^13^C-PFBS
L-PFHxS	Perfluorohexanesulfonic acid (branched and linear)	355-46-4	Br-PFHxSK, 40.55 mg L^−1^	Br-PFHxSK, 40.55 mg L^−1^	^13^C-PFHxS
B-PFHxS			Br-PFHxSK, 9.45 mg L^−1^	Br-PFHxSK, 9.45 mg L^−1^	^13^C-PFHxS
PFHpS	Perfluoroheptanesulfonic acid	375-92-8	L-PFHpS, 50 mg L^−1^	L-PFHpS, 50 mg L^−1^	^13^C-PFHxS
L-PFOS	Perfluorooctanesulfonic acid	1763-23-1	L-PFOS, 50 mg L^−1^	L-PFOS, 50 mg L^−1^	^13^C-PFOS
B-PFOS	Sodium perfluoro-5-methylheptanesulfonate Sodium perfluoro-5,5-dimethylhexanesulfonate		P5MHpS, 1mg L^−1^ P55DMHxS, 1mg L^−1^	P5MHpS, 1mg L^−1^ P55DMHxS, 1mg L^−1^	^13^C-PFOS
PFNS	Perfluorononanesulfonic acid	68259-12-1	L-PFNS, 50 mg L^−1^	L-PFNS, 50 mg L^−1^	^13^C-PFOS
PFDS	Perfluorodecanesulfonic acid	335-77-3	L-PFDS, 50 mg L^−1^	L-PFDS, 50 mg L^−1^	^13^C-PFOS
4:2 FTS	1H, 1H, 2H, 2H, perfluorohexane sulfonic acid	757124-72-4	4:2FTS, 50 mg L^−1^	4:2FTS, 50 mg L^−1^	^13^C-4:2FTS
6:2 FTS	1H, 1H, 2H, 2H, perfluorooctane sulfonic acid	27619-97-2	6:2FTS, 50 mg L^−1^	6:2FTS, 50 mg L^−1^	^13^C-6:2FTS
8:2 FTS	1H, 1H, 2H, 2H, perfluorodecane sulfonic acid	39108-34-4	8:2FTS, 50 mg L^−1^	8:2FTS, 50 mg L^−1^	^13^C-8:2FTS
MeFOSAA	N-Methylperfluorooctane sulfonamidoacetic acid	2355-31-9	Br-NMeFOSAA, 50 mg L^−1^	N-MeFOSAA, 50 mg L^−1^	^2^H-MeFOSAA
EtFOSAA	N-Ethylperfluorooctane sulfonamidoacetic acid	2991-50-6	Br-NEtFOSAA, 50 mg L^−1^	N-EtFOSAA, 50 mg L^−1^	^2^H-EtFOSAA
PFBSA	Perfluorobutanesulfonamide	30334-69-1	FBSA-I, 50 mg L^−1^	FBSA-I, 50 mg L^−1^	^13^C-HFPO-DA
PFHxSA	Perfluorohexanesulfonamide	41997-13-1	FHxSA-I, 50 mg L^−1^	FHxSA-I, 50 mg L^−1^	^13^C-PFOS
PFOSA	Perfluorooctanesulfonamide	754-91-6	FOSA-I, 50 mg L^−1^	FOSA-I, 50 mg L^−1^	^13^C-PFOSA
PFecHS	Potassium Perfluoro-4-ethylcyclohexanesulfonate	67584-42-3	PFECHS, 50 mg L^−1^	PFECHS, 50 mg L^−1^	^13^C-PFOS
NFDHA	Nonafluoro-3,6-dioxaheptanoic acid	151772-58-6	3,6-OPFHpA, 50 mg L^−1^	PFAC-MXG, 2 mg L^−1^	^13^C-PFHxA
PFEESA	Perfluoro (2-ethoxyethane) sulfonic acid	113507-82-7	PFEESA, 50 mg L^−1^	PFAC-MXG, 2 mg L^−1^	^13^C-PFBS
PFMPA	Perfluoro-3-methoxypropanoic acid	377-73-1	PF4OPeA, 50 mg L^−1^	PFAC-MXG, 2 mg L^−1^	^13^C-PFPeA
PFMBA	Perfluoro-4-methoxybutanoic acid	863090-89-5	PF5OHxA, 50 mg L^−1^	PFAC-MXG, 2 mg L^−1^	^13^C-PFBS
HFPO-DA	Hexafluoropropylene oxide dimer acid	13252-13-6	HFPO-DA, 50 mg L^−1^	PFAC-MXF, 2 mg L^−1^	^13^C-HFPO-DA
ADONA	Dodecafluoro-3H-4,8-dioxanonanoate	919005-14-4	NaDONA, 50 mg L^−1^	PFAC-MXF, 2 mg L^−1^	^13^C-PFHxS
9Cl-PF3ONS	9-chlorohexadecafluoro-3-oxanonane-1-sulfonate	756426-58-1	9Cl-PFONS, 50 mg L^−1^	PFAC-MXF, 2 mg L^−1^	^13^C-PFOS
11Cl-PF3OUdS	11-chloroeicosafluoro-3-oxaundecane-1-sulfonate	763051-92-9	11Cl-PFOUdS, 50 mg L^−1^	PFAC-MXF, 2 mg L^−1^	^13^C-PFDoA

**Table 2:** Accuracy/Recovery and Precision.

	Imprecision, Inter-Day (Internal PT Samples)	Imprecision, Inter-Day (Quality Control Samples)	Imprecision, Intra-Day (Internal PT Samples)	Accuracy, (Internal PT Samples)	Accuracy, (Quality Control Samples)
PFBA	11.2%	14.1%	8.0%	89.5%	93.5%
PFPeA *	14.6%	13.6%	12.7%	87.3%	87.8%
PFHxA *	13.8%	12.9%	9.4%	91.5%	91.4%
PFHpA *	10.9%	14.3%	7.6%	90.5%	93.8%
L-PFOA	14.0%	15.5%	11.4%	92.6%	90.7%
B-PFOA	27.4%	13.8%	25.4%	81.4%	87.6%
PFNA	14.0%	13.5%	7.5%	83.1%	92.6%
PFDA	15.2%	13.8%	11.3%	86.7%	91.9%
PFUnA	15.4%	11.6%	11.7%	90.9%	90.2%
PFDoA	16.4%	13.2%	12.3%	92.8%	90.3%
PFTriA	18.4%	15.6%	14.3%	92.1%	88.2%
PFTeA	16.9%	13.8%	12.6%	91.2%	88.0%
PFPrS	13.6%	10.4%	11.4%	86.3%	89.8%
PFBS	11.3%	8.6%	8.0%	92.0%	94.0%
PFPeS	9.9%	9.6%	9.3%	91.4%	86.8%
L-PFHxS	9.4%	8.8%	6.1%	94.0%	90.4%
B-PFHxS	14.1%	9.2%	8.5%	92.6%	95.3%
PFHpS	16.1%	11.3%	13.2%	81.3%	88.4%
L-PFOS	13.3%	8.8%	10.6%	87.8%	90.8%
B-PFOS	14.9%	11.0%	12.9%	86.6%	81.9%
PFNS	12.2%	12.1%	9.5%	93.5%	92.2%
PFDS	16.8%	13.8%	13.6%	94.6%	92.4%
4:2 FTS	12.1%	10.5%	8.9%	89.7%	90.6%
6:2 FTS *	13.9%	9.9%	7.1%	89.6%	92.7%
8:2 FTS	14.3%	8.5%	10.6%	85.2%	89.3%
MeFOSAA	11.0%	11.1%	8.3%	83.5%	89.8%
EtFOSAA	12.7%	13.1%	9.0%	86.8%	91.0%
PFBSA	18.4%	11.6%	17.0%	92.4%	86.5%
PFHxSA	17.3%	13.4%	14.5%	96.2%	81.8%
PFOSA	11.4%	11.5%	9.0%	89.1%	83.8%
PFECHS	15.2%	12.8%	12.8%	88.4%	90.5%
NFDHA	9.4%	10.3%	7.2%	90.3%	83.5%
PFEESA	10.7%	9.1%	7.9%	91.6%	85.7%
PFMPA	14.4%	10.1%	12.1%	91.6%	83.7%
PFMBA	13.1%	10.9%	10.9%	93.2%	84.0%
HFPO-DA	13.0%	11.3%	9.8%	90.8%	90.1%
ADONA	13.5%	11.3%	11.1%	85.0%	90.3%
9Cl-PF3ONS	12.7%	11.6%	10.5%	89.7%	91.3%
11Cl-PF3OUdS	16.3%	13.9%	13.0%	87.8%	87.3%

* Analyte had variability at the lowest levels due to contamination present within the cards; these highly variable values were not included in the averages shown.

**Table 3:** Sensitivity (LOD/LOQ), Reportable Range Accuracy, Normalized Percent Matrix Bias. Further calculations regarding matrix studies can be found in Table S1.

	SD/m (S0)	LOD (3S0)	LOQ (10S0)	S1	Reportable Range Accuracy	Normalized Percent Matrix Bias
PFBA	0.0050	0.0149	0.0498	0.050	80.1–99.0%	0.2%
PFPeA	0.0047	0.0141	0.0469	0.050	80.0–98.9%	3.4%
PFHxA	0.0048	0.0143	0.0477	0.050	81.5–99.4%	7.7%
PFHpA	0.0043	0.0130	0.0432	0.050	82.4–99.1%	1.7%
L-PFOA	0.0045	0.0135	0.0449	0.050	74.5–99.3%	8.2%
B-PFOA	0.0049	0.0148	0.0494	0.050	85.0–100%	2.6%
PFNA	0.0048	0.0145	0.0482	0.050	85.8–99.2%	11.0%
PFDA	0.0048	0.0145	0.0483	0.050	82.0–98.9%	9.1%
PFUnA	0.0049	0.0146	0.0487	0.050	84.1–99.1%	5.4%
PFDoA	0.0048	0.0143	0.0478	0.050	77.5–99.3%	4.6%
PFTriA	0.0049	0.0146	0.0488	0.050	82.1–98.7%	11.0%
PFTeA	0.0046	0.0137	0.0458	0.050	77.6–99.1%	1.2%
PFPrS	0.0032	0.0097	0.0324	0.050	75.1–99.3%	6.9%
PFBS	0.0036	0.0107	0.0355	0.050	80.2–99.3%	8.3%
PFPeS	0.0030	0.0090	0.0300	0.050	86.0–98.3%	2.5%
L-PFHxS	0.0024	0.0071	0.0237	0.041	84.7–99.2%	2.0%
B-PFHxS	0.0007	0.0022	0.0074	0.009	86.8–100%	2.5%
PFHpS	0.0032	0.0095	0.0316	0.050	84.2–98.5%	5.5%
L-PFOS	0.0031	0.0094	0.0312	0.050	81.4–99.1%	3.1%
B-PFOS	0.0038	0.0114	0.0381	0.050	88.9–99.9%	1.4%
PFNS	0.0040	0.0120	0.0401	0.050	75.4–99.3%	6.5%
PFDS	0.0044	0.0133	0.0443	0.050	82.1–99.4%	4.3%
4:2 FTS	0.0041	0.0124	0.0413	0.050	76.9–98.6%	5.5%
6:2 FTS	0.0049	0.0148	0.0493	0.050	77.7–98.5%	2.3%
8:2 FTS	0.0034	0.0102	0.0339	0.050	70.9–98.0%	4.0%
MeFOSAA	0.0033	0.0098	0.0328	0.050	83.4–98.9%	3.6%
EtFOSAA	0.0039	0.0118	0.0394	0.050	81.0–99.3%	1.4%
PFBSA	0.0046	0.0137	0.0456	0.050	75.7–99.3%	10.6%
PFHxSA	0.0050	0.0149	0.0496	0.050	74.5–98.5%	5.7%
PFOSA	0.0035	0.0106	0.0353	0.050	85.0–99.2%	8.9%
PFECHS	0.0039	0.0118	0.0394	0.050	76.0–99.4%	6.5%
NFDHA	0.0037	0.0110	0.0366	0.050	88.3–98.9%	5.3%
PFEESA	0.0029	0.0086	0.0285	0.050	87.0–98.5%	1.4%
PFMPA	0.0031	0.0094	0.0314	0.050	87.4–99.1%	6.5%
PFMBA	0.0035	0.0104	0.0347	0.050	84.7–98.2%	2.4%
HFPO-DA	0.0036	0.0108	0.0361	0.050	78.7–99.0%	6.5%
ADONA	0.0033	0.0098	0.0327	0.050	74.4–98.7%	4.2%
9Cl-PF3ONS	0.0050	0.0150	0.0499	0.050	74.0–99.0%	4.3%
11Cl-PF3OUdS	0.0047	0.0141	0.0470	0.050	80.7–98.9%	6.5%

**Table 4:** Method Interferences for TDCA and high-POP samples. Each value shown is a percent difference as compared to the expected analyte value.

	4.983 mg L^−1^ TDCA	49.83 mg L^−1^ TDCA	POP Level 1	POP Level 2
PFBA	6%	−15%	−4%	1%
PFPeA	8%	−13%	−4%	0%
PFHxA	9%	−10%	1%	4%
PFHpA	7%	−14%	0%	3%
L-PFOA	5%	−16%	−11%	−6%
B-PFOA	6%	−16%	2%	4%
PFNA	6%	−16%	0%	−1%
PFDA	7%	−18%	−6%	−2%
PFUnA	11%	−12%	−2%	1%
PFDoA	6%	−22%	−2%	2%
PFTriA	4%	−14%	1%	7%
PFTeA	1%	−16%	3%	5%
PFPrS	9%	−17%	−2%	4%
PFBS	6%	−19%	19%	−1%
PFPeS	7%	−18%	1%	0%
L-PFHxS	8%	−17%	−13%	−10%
B-PFHxS	10%	−12%	−3%	0%
PFHpS	8%	−13%	−5%	−2%
L-PFOS	6%	−9%	−3%	−7%
B-PFOS	4%	−10%	−10%	−12%
PFNS	6%	−14%	0%	7%
PFDS	5%	−14%	−3%	2%
4:2FTS	6%	−19%	2%	−3%
6:2FTS	9%	−13%	−12%	−17%
8:2FTS	6%	−19%	−7%	1%
MeFOSAA	5%	−18%	0%	5%
EtFOSAA	9%	−12%	2%	−2%
PFOSA	5%	−12%	−3%	4%
PFECHS	3%	−16%	−1%	3%
NFDHA	7%	−17%	0%	2%
PFEESA	7%	−20%	−1%	−2%
PFMPA	8%	−17%	−2%	3%
PFMBA	5%	−20%	−3%	−3%
HFPO-DA	6%	−19%	−1%	0%
ADONA	7%	−16%	−2%	0%
9Cl-PF3ONS	1%	−18%	0%	4%
11Cl-PF3OUdS	6%	−13%	−5%	−2%

**Table 5:** Maximum Dilutions. Each value shown is a percent difference as compared to the expected analyte value using the pre-extraction dilution method, the post-extraction dilution method, or the post-extraction dilution method using an ISTD “Correction Factor”.

	Pre-Extraction Dilution Average % Difference	Post-Extraction Dilution Average % Difference	ISTD “Correction Factor” Corrected Post-Extraction Dilution Average % Difference
PFBA	8%	10%	7%
PFPeA	7%	15%	6%
PFHxA	6%	15%	8%
PFHpA	7%	16%	9%
L-PFOA	6%	17%	4%
B-PFOA	7%	18%	3%
PFNA	7%	15%	7%
PFDA	7%	15%	4%
PFUnA	6%	17%	2%
PFDoA	8%	9%	14%
PFTriA	8%	7%	16%
PFTeA	7%	13%	4%
PFPrS	7%	13%	9%
PFBS	5%	15%	9%
PFPeS	5%	16%	8%
L-PFHxS	6%	16%	9%
B-PFHxS	4%	17%	8%
PFHpS	6%	12%	13%
L-PFOS	8%	12%	9%
B-PFOS	6%	17%	4%
PFNS	7%	9%	12%
PFDS	6%	14%	8%
4:2 FTS	7%	13%	33%
6:2 FTS	9%	12%	21%
8:2 FTS	11%	7%	11%
MeFOSAA	8%	11%	15%
EtFOSAA	7%	13%	16%
PFOSA	8%	90%	100%
PFECHS	6%	17%	4%
NFDHA	5%	17%	5%
PFEESA	6%	15%	9%
PFMPA	8%	9%	12%
PFMBA	6%	13%	11%
HFPO-DA	7%	15%	7%
ADONA	6%	15%	10%
9Cl-PF3ONS	8%	13%	8%
11Cl-PF3OUdS	5%	14%	9%

## Data Availability

The original contributions presented in this study are included in the article/Supplementary Materials. Further inquiries can be directed to the corresponding author.

## Supplementary Materials

The following supporting information can be downloaded at: https://www.mdpi.com/article/10.3390/toxics14090753/s1. Table S1: Additional Matrix Effects Information. Normalized Percent Matrix Bias, Matrix Factor (MF), Internal Standard (IS) Normalized MF, and the raw coefficient of variation (%CV) across all 8 lots of matrix tested.
