# Supplementary material for: Per- and Polyfluoroalkyl Substances (PFAS) in Michigan: A Novel High-Throughput Method with Liquid Chromatography Tandem Mass Spectrometry (LC-MS/MS) Analysis of 39 PFAS Compounds in Human and Bovine Dried Blood Spots (DBS)
Source: Toxics. 2026 Aug 26;14(9):753. doi: 10.3390/toxics14090753 (PMC13611127; doi:10.3390/toxics14090753)
Supplement: Supplementary file 1 [file toxics-14-00753-s001.zip › toxics-4480656-supplementary.pdf]

Table S1. Additional Matrix Effects Information. Normalized Percent Matrix Bias, Matrix Factor (MF), Internal Standard (IS) Normalized MF, and the raw coefficient of variation (%CV) across all 8 lots of matrix tested.

Component Name	Normalized Percent Matrix Bias	Matrix Factor	IS Normalized MF	%CV (8 lots)
11Cl-PF3OUdS	6.5%	0.9	1.1	8%
4:2FTS	5.5%	1.3	1.1	12%
6:2FTS	2.3%	1.1	1.1	11%
8:2FTS	4.0%	0.8	1.1	8%
9Cl-PF3ONS	4.3%	0.9	1.1	10%
ADONA	4.2%	0.9	1.1	9%
Br-PFHxS	2.5%	0.9	1.1	11%
Br-PFOA	2.6%	0.9	1.1	9%
Br-PFOS	1.4%	0.8	1.0	8%
EtFOSAA	1.4%	1.0	1.1	10%
HFPO-DA	6.5%	0.9	1.1	11%
L-PFHxS	2.0%	0.8	1.1	10%
L-PFOA	8.2%	0.9	1.1	9%
L-PFOS	3.1%	0.8	1.0	8%
MeFOSAA	3.6%	0.9	1.1	9%
NFDHA	5.3%	0.9	1.1	11%
PFBA	0.2%	0.9	1.1	9%
PFBS	8.3%	0.9	1.1	10%
PFBSA	10.6%	0.9	1.1	11%
PFDA	9.1%	0.9	1.1	7%
PFDoA	4.6%	0.8	1.1	8%
PFDS	4.3%	0.8	1.0	9%
PFECHS	6.5%	0.8	1.1	9%
PFEESA	1.4%	0.9	1.1	10%
PFHpA	1.7%	0.9	1.1	11%
PFHpS	5.5%	0.8	1.1	10%
PFHxA	7.7%	0.9	1.1	9%
PFHxSA	5.7%	0.9	1.1	9%
PFMBA	2.4%	0.9	1.1	10%
PFMPA	6.5%	0.9	1.1	11%
PFNA	11.0%	0.8	1.1	8%
PFNS	6.5%	0.9	1.1	9%
PFOSA	8.9%	0.9	1.1	9%
PFPeA	3.4%	0.9	1.1	10%
PFPeA	2.5%	0.9	1.1	10%
PFPrS	6.9%	0.9	1.1	11%
PFTeA	1.2%	0.8	1.1	8%

PFTriA	11.0%	0.9	1.2	7%
PFUnA	5.4%	0.9	1.1	9%
